# A Structural Landscape of Neutralizing Antibodies Against SARS-CoV-2 Receptor Binding Domain

**DOI:** 10.3389/fimmu.2021.647934

**Published:** 2021-04-28

**Authors:** Ling Niu, Kathryn N. Wittrock, Gage C. Clabaugh, Vikram Srivastava, Michael W. Cho

**Affiliations:** ^1^ Department of Biomedical Sciences, College of Veterinary Medicine, Iowa State University, Ames, IA, United States; ^2^ NeoVaxSyn, Inc. Ames, IA, United States

**Keywords:** SARS-CoV-2, RBD, COVID-19, neutralizing antibody, neutralizing epitope

## Abstract

SARS-CoV-2, the novel coronavirus responsible for the ongoing COVID-19 pandemic, has been spreading rampantly. The global scientific community has responded rapidly to understand immune correlates of protection to develop vaccines and immunotherapeutics against the virus. The major goal of this mini review is to summarize current understanding of the structural landscape of neutralizing antibodies (nAbs) that target the receptor binding domain (RBD) of viral spike (S) glycoprotein. The RBD plays a critical role in the very first step of the virus life cycle. Better understanding of where and how nAbs bind the RBD should enable identification of sites of vulnerability and facilitate better vaccine design and formulation of immunotherapeutics. Towards this goal, we compiled 38 RBD-binding nAbs with known structures. Review of these nAb structures showed that (1) nAbs can be divided into five general clusters, (2) there are distinct non-neutralizing faces on the RBD, and (3) maximum of potentially four nAbs could bind the RBD simultaneously. Since most of these nAbs were isolated from virus-infected patients, additional analyses of vaccine-induced nAbs could facilitate development of improved vaccines.

## Introduction

Just over a century after the 1918 flu pandemic, humanity is experiencing another major pandemic. The COVID-19 pandemic, which is caused by SARS-CoV-2, began in late 2019. In just over one year of the pandemic, about 118 million people have been infected with the virus and, despite advanced modern medicine, over 2.6 million have died. COVID-19 is likely to pose a continuing threat to the global economy and public health systems worldwide unless most of the population is vaccinated.

SARS-CoV-2 is classified as a *betacoronavirus* of the *Coronaviridae* family. The virus is closely related to a bat coronavirus, RaTG13, with nucleotide sequence identity of ~96% (1). It is also distantly related to SARS-CoV (82% identity), the virus that caused the 2002-2003 SARS epidemic. Both viruses use angiotensin converting enzyme 2 (ACE2) as a receptor (1). Binding to ACE2 and virus entry into host cells are mediated by spike (S) glycoprotein. Because of the high similarity, past research on SARS-CoV likely facilitated a rapid response to SARS-CoV-2, allowing for quicker development of vaccines and treatments.

During the past year, there have been unprecedented global efforts to develop vaccines against the virus. Already, multiple vaccine candidates have either completed or almost completed their Phase 3 clinical trials. Two of them, by Moderna (2) and Pfizer/BioNTech (3), have been shown to be ~95% effective. Both of them have been approved by the U.S. FDA for emergency use. Although these vaccines have been shown to be effective in the short-term, their long-term efficacy has not yet been demonstrated. Thus, continued evaluation of immune correlates of protection and characterization of antigenic and immunogenic properties of S glycoprotein are needed to develop more efficacious vaccines in the future.

Neutralizing antibodies (nAbs) play a critical role in providing protective immunity against viral diseases. Recently, it’s been shown that 90% of nAbs mounted against SARS-CoV-2 in COVID-19 patients target the receptor binding domain (RBD) of S glycoprotein (4). Better understanding of their potency and how they bind their target epitopes could allow for the design of more effective vaccines and improve immunotherapeutic agents. During the past several months, many neutralizing monoclonal antibodies (mAbs) against the RBD have been isolated and their structures have been solved using Cryo-EM or X-Ray Crystallography. An excellent review article was published on this topic (5). Since then, many more nAbs have been isolated and their structures have been solved. Considering the rapid progress being made in this field, and the many new viral variants with different RBD mutations emerging, we felt a mini review with more up-to-date information would be beneficial to many investigators, especially to those who work on immunogen design for vaccine development. In this review, we have compiled IC_50_ values of all nAbs with known structures and identified key amino acid residues targeted by them. Superimposing all nAbs revealed clusters of nAbs and non-neutralizing faces on the RBD.

## Neutralizing Antibodies Against the RBD With Known Structures

**Figure 1:**
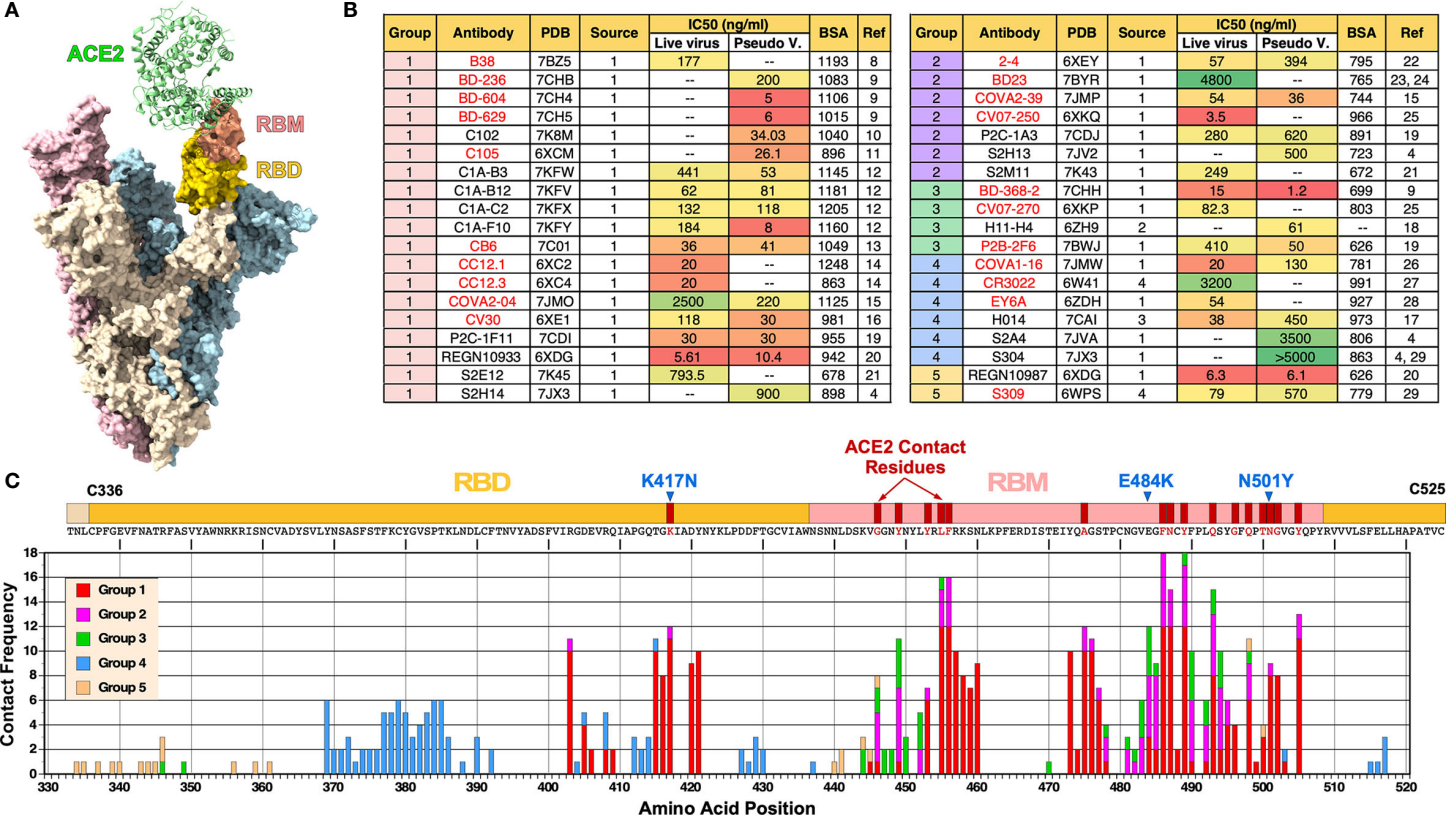
Summary of nAbs against SARS-CoV-2 RBD. **(A)** Cryo-EM structure of trimeric spike glycoprotein (PDB: 7A97). One of the monomers (beige) with ACE2 bound has its RBD and RBM colored in gold and salmon, respectively. **(B)** Antibodies are organized by their cluster group. Antibody source (1: COVID-19 patients; 2: Llama antibody library; 3: SARS-CoV RBD immunized mice; 4: SARS-CoV patients), IC_50_ and buried surface area (BSA) are shown. IC_50_ was converted from nM using ~150 kd molecular weight for the following mAbs: H014, REGN10933, REGN10987, S2M11S2E12, S309. BSA values were either obtained from a previous review (5) or calculated using PISA (6). Antibodies shown in red text have been previously described (5). **(C)** Frequency of nAbs making contact with each amino acid in the RBD. Each group is represented by different colors. Contact residues were identified using “Clashes/Contacts” tool in UCSF Chimera using default settings. Three RBD mutations present in SARS-CoV-2 B.1.351 variant (K417N, E484K and N501Y) are shown.

The S glycoprotein functions as a trimer (**Figure 1A**). The RBD within S glycoprotein is structurally defined as a region between two cysteine residues (C^336^ and C^525^) that form a disulfide bridge. Within the RBD, there is a short linear segment called receptor binding motif (RBM) that contains most of the amino acid residues that make contact with ACE2.

According to CoV-AbDab database (http://opig.stats.ox.ac.uk/webapps/covabdab/) (7), there are 413 neutralizing mAbs and 100 nanobodies that bind SARS-CoV-2 RBD (as of December 27, 2020). Of these, we have identified the following 38 mAbs, for which high resolution structures have been determined with the RBD: B38 (8), BD-236, BD-368-2, BD-604, BD-629 (9), C102 (10), C105 (11), C1A-B3, C1A-B12, C1A-C2, C1A-F10 (12), CB6 (13), CC12.1, CC12.3 (14), COVA2-04, COVA2-39 (15), CV30 (16), H014 (17), H11-H4 (18), P2B-2F6, P2C-1F11, P2C-1A3 (19), REGN10933, REGN10987 (20), S2E12, S2M11 (21), S2A4, S2H13, S2H14, S304 (4), 2-4 (22), BD23 (23, 24), CV07-250, CV07-270 (25), COVA1-16 (26), CR3022 (27), EY6A (28), and S309 (29). The Protein Data Bank (PDB) ID, antibody source, half-maximal inhibitory concentrations (IC_50_), and total buried surface area (BSA-Å^2^) of these nAbs are summarized on **Figure 1B**. BSA was calculated using PISA program (6).

**Figure 2:**
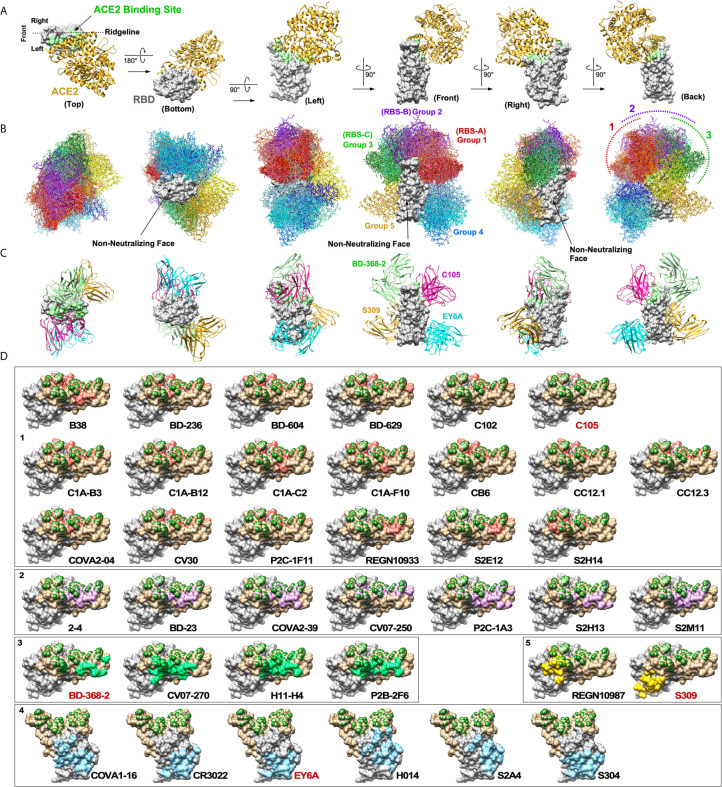
Neutralizing antibodies against SARS-CoV-2 RBD. **(A)** A cocrystal structure of the RBD (grey) bound to ACE2 (gold) is shown in six orientations (PDB: 6M0J). ACE2 binding site is highlighted in light green. **(B)** Structures of 38 nAbs were superimposed onto the RBD. Five groups of nAbs are shown in different color tones (1: red, 2: purple, 3: green, 4: blue and 5: yellow). Three non-neutralizing faces are indicated. **(C)** Maximum of up to four nAbs could potentially bind the RBD simultaneously. **(D)** Footprints of individual nAbs are shown. To generate footprints, “Clashes/Contacts” tool in UCSF Chimera was used to identify residues on RBD that contact nAbs. Default contact criteria of VDW overlap ≥ -0.4 Å was used. ACE2 contact residues are shown in dark green. Footprints are colored as for panel **(B)**.

The antibodies are organized based on their clustering groups (Groups 1-5), which is defined by where on RBD they bind (see **Figure 2** and explanations below). Most nAbs were isolated from COVID-19 patients. In contrast, CR3022 (27) and S309 (29) are cross reactive antibodies isolated from SARS-CoV-infected individuals and H014 was isolated from a phage antibody library generated from a mouse immunized with SARS-CoV RBD (17). H11-H4 was isolated from a llama antibody library (18).

Neutralizing activity of mAbs were assessed by using either live, infectious SARS-CoV-2 or pseudoviruses or both. Their IC_50_ values (in ng/ml) are shown in **Figure 1B**. Overall, we were not able to see a clear correlation between clustering groups and neutralization potency. Furthermore, there was no consistency between IC_50_ determined using live virus vs. pseudoviruses. While IC_50_ was significantly higher using live viruses for some antibodies (*e.g*., C1A-B3, C1A-F10, COVA2-04), for other antibodies, IC_50_ was higher using pseudoviruses (*e.g*., 2-4, H014, S309). For some antibodies, the values were more similar (*e.g*., C1A-B12, P2C-1F11, COVA2-39). These differences and inconsistencies could be due to the fact that different laboratories are using different pseudoviruses (*e.g*., VSV-based or lentivirus-based) or differences in how live virus neutralization assays were done (*e.g*., amount of virus used, or the duration of virus incubated with antibodies prior to adding to cells). In this regard, the field must standardize virus neutralization assays to compare the relative potency of different nAbs and also to compare the relative efficacy of different vaccines.

Clearly, there are more nAbs in Group 1 than other groups, especially Groups 3 and 5. Assuming that the process of isolating nAbs was random, this could suggest that epitopes targeted by Group 1 nAbs are more immunogenic than other epitopes or that antibodies that target Group 1 epitopes are more likely to exhibit neutralizing activity or both.

The frequency of amino acid residues on the RBD that make contact with nAbs is shown in **Figure 1C**. Based on this analysis, the ten most frequently targeted residues are F^486^, Y^489^, L^455^, F^456^, N^487^, Q^493^, Y^505^, K^417^, A^475^ and E^484^. All of these residues are situated within the RBM except for K^417^, which lies outside of the RBM but makes contact with ACE2. Not surprisingly, SARS-CoV-2 B.1.351 variant (*a.k.a*. 20H/501Y.V2 South Africa strain) that has K417N and E484K mutations in addition to N501Y has been shown to resist neutralization by vaccine-induced nAbs (30).

## Structural Landscape of Nabs Against the RBD

A cocrystal structure of the RBD bound to ACE2 has been solved (31). The structure is shown from six different perspectives with ACE2 positioned above the RBD (**Figure 2A**). For orientation, the view from the “back” represents ACE2 bound to a trimeric spike shown in **Figure 1A**. A view from the top shows that ACE2 binds mainly on one side of the ridge (arbitrarily defined as the “left side”). To assess structural landscape of nAbs against the RBD, we superimposed 38 nAbs onto the RBD (**Figure 2B**). The antibodies were categorized into five groups (1 through 5) based on how they clustered together and how they bound the RBD. The five groups are shown in different colors. Group 1 nAbs (red tone) bind the receptor binding site (RBS) very much like ACE2 on the “left side” of the ridge. The top five most targeted amino acid residues by Group 1 nAbs are L^455^, F^456^, F^486^, N^487^ and Y^489^. Group 2 nAbs (purple tone) also bind the RBS, but they are positioned more upright and straddle on the center of the ridge. The top three most targeted amino acid residues by Group 2 nAbs are Y^449^, G^485^ and F^486^. Group 3 nAbs (green tone) bind on the “right side” of the ridge opposite from Group 1 nAbs. The top three most targeted amino acid residues by Group 3 nAbs are Y^449^, E^484^ and F^490^. These three groups have been referred to as RBS-A, -B and -C, respectively, in the previous review (5). The mechanism of neutralization by these three groups of nAbs is mainly through direct competition with ACE2 for binding the RBD. However, the degree of overlap between Group 3 nAbs and ACE2 is much less than Group 1 and Group 2 nAbs. Group 4 nAbs (blue tone) bind the lower half of the “left side” of the RBD. The top four most targeted amino acids by Group 4 nAbs are Y^369^, C^379^, P^384^ and T^385^. This group of nAbs has been previously referred to as “CR3022 cryptic site” after the prototypic antibody (5). While four of these antibodies are able to block binding of ACE2 to the RBD (COVA1-16, H014, S2A4 and partially by S304), CR3022 and EY6A do not. Instead, the neutralization mechanism of the latter two nAbs is thought to be by destabilizing prefusion conformation of the trimeric spike (28, 32). Group 5 nAbs (yellow tone) bind “rear right” side of the protein. While REGN10987 can block binding of ACE2 to the RBD, S309 cannot. Although the neutralization mechanism of S309 is not clear, it is thought that a part of its activity could be cross-linking of the trimeric structure, steric hindrance of conformational changes or aggregation of virions (29).

By compiling all nAb structures onto the RBD, what became evident are non-neutralizing faces (NNF) where no known nAbs bind (“bottom”, “front” and front half of the “right”). Although it is possible that nAbs that bind these regions simply have not yet been isolated, this is highly unlikely considering the number of nAbs that have already been characterized. Rather, it is more likely that antibodies that bind these regions neither block ACE2 binding nor prevent conformational changes that are required for membrane fusion. It is also possible that these regions might be non-immunogenic because they are not accessible to antibodies in the context of trimeric S glycoprotein complexes. The existence of these NNF is important from a vaccine development standpoint, especially for designing RBD-based immunogens, because if they are highly immunodominant, high levels of non-neutralizing antibodies against the NNF could sterically interfere with induction of nearby nAbs.

For developing immunotherapeutics, it is desirable to use a cocktail of multiple nAbs rather than using only one to minimize the chance of giving rise to neutralization-resistant mutant variants. Our analyses show that a total of up to four nAbs could potentially bind a single RBD at the same time (Groups 1, 3, 4 and 5). Four nAbs bound to RBD are shown in **Figure 2C**: C105, BD-368-2, EY6A and S309. Except for a minor clash between S309 and K76 residue on the heavy chain of BD-368-2, all four nAbs could bind at the same time. Because of clashes between Group 2 nAbs and those of Groups 1 and 3, only three nAbs would be able to bind the RBD if a Group 2 nAb was used. Also, not all Group 1 nAbs could bind with all Group 3 nAbs. It is important to note that full length IgG molecules are much bulkier than Fab and that there are other neighboring RBDs in a trimeric spike complex. This could limit how many nAbs can actually bind a single RBD at the same time. However, it is also important to keep in mind that multiple nAbs do not have to bind the same RBD simultaneously to exert synergy. As long as multiple nAbs can bind a single virus particle simultaneously, a cocktail immunotherapy should be able to reduce the chances of giving rise to neutralization-escape mutants. Current therapy by Regeneron uses REGN10933 (Group 1) and REGN10987 (Group 5) (33). For side-by-side comparison, footprints of 38 nAbs are shown individually in **Figure 2D**.

## Discussion

The structural overview of nAbs against the RBD provides three-dimensional details of how they bind the antigen as well as insights into potential mechanisms of their action. This information is highly valuable for formulating effective immunotherapeutics. Although the structural data we compiled in this review provides a good visual representation of the nAb landscape, additional information on the immunological properties of the RBD is needed for designing improved vaccines. Specifically, information on the relative immunogenicity of different neutralizing and non-neutralizing epitopes is needed. The immunodominance of particular epitopes will likely depend on not only the genotype of the individuals, but also the immunogens. Both the Pfizer and Moderna vaccines are based on the full-length trimeric S protein complexes that are expressed on the membrane surface, similar to the antigens to which virus-infected patients would be exposed. It would be interesting to compare the nAbs elicited by these vaccines to those isolated from virus-infected patients, as well as to those induced by other immunogens (*e.g.*, soluble RBD).

Global establishment of new viral lineages of SARS-CoV-2 has been reported recently (34, 35). Accordingly, one of the major concerns with the COVID-19 pandemic is the emergence of SARS-CoV-2 variants that would become resistant to current neutralizing monoclonal antibody (mAb) therapies or those that can elude immune responses elicited by currently available vaccines. One possible solution is to predict potential neutralization-escape variants and preemptively design and develop new vaccines or therapeutics. Recently, RBD mutations that enable viruses to escape neutralization by REGN-COV2 cocktail (mAbs REGN10933 and REGN10987) and LY-CoV016 (*a.k.a* CB6) were mapped using a library of RBD variants (36). As expected, escape mutations largely appeared in the antibody-RBD interface. One intriguing exception is that E406W mutation, which enables escape from neutralization by both REGN10933 and REGN10987, is not in direct contact with either antibody. Comparison of experimentally identified escape mutations and available sequences of currently circulating SARS-CoV-2 indicated that many viral variants that can resist one or more nAbs already exist. However, the only variant present in >0.1% of sequences were the REGN10933 escape mutant Y453F, the REGN10987 escape-mutant N439K and the CB6 escape-mutant K417N (36).

Mutations in the RBD that affect recognition by polyclonal human plasma antibodies were also mapped (37). These mutations map largely to three distinct regions: (i) “Front” end of the ridge near F^456^ and E^484^, (ii) a loop spanning S^443^-N^450^ and an adjacent S^494^-N^501^ region near where REGN10987 binds at the “back” end of the ridge, and (iii) a patch near P^384^ where Group 4 nAbs bind. The most important site was shown to be E484, mutations of which reduced neutralizing activity of some plasma by >10-fold. More than 0.1% of all sequenced isolates have mutations at E484 (37). It is interesting to note that our analysis shows that nAbs from Groups 1, 2 and 3 recognize E484 with similar frequency (**Figure 1C**).

It is highly likely that SARS-CoV-2 will persist in the human population for the foreseeable future, especially if not everyone is vaccinated within a short period of time. We expect the virus will continue to mutate, adapt and evolve to survive as a species, just like any other living organism on earth. Eventually, humans and SARS-CoV-2 may have to find a way to coexist without killing each other, as has happened with many other viruses.

## Author Contributions

MC: Supervised writing, analyzed all data, generated the final figure and table, and did the final editing. LN: Generated figures using UCSF Chimera, and participated in generating the table and writing the manuscript. KW: Drafted the manuscript and participated in generating the table. GC: Assisted in writing the manuscript and participated in generating the table. VS: Participated in analyzing data and assisted in writing the manuscript. All authors contributed to the article and approved the submitted version.

## Funding

This work was funded by Iowa State University. Publication fee is also provided by the university.

## Conflict of Interest

MC has an equity interest in NeoVaxSyn Inc. and serves as the CEO/President. NeoVaxSyn did not contribute to this work or the interpretation of the data.

The remaining authors declare that the research was conducted in the absence of any commercial or financial relationships that could be construed as a potential conflict of interest.

## References

[1] Zhou P Yang XL Wang XG Hu B Zhang L Zhang W. A Pneumonia Outbreak Associated With a New Coronavirus of Probable Bat Origin. Nature (2020) 579(7798):270–3. 10.1038/s41586-020-2012-7 PMC7095418 32015507

[2] Baden LR El Sahly HM Essink B Kotloff K Frey S Novak R. Efficacy and Safety of the Mrna-1273 SARS-CoV-2 Vaccine. N Engl J Med (2021) 384(5):403–16. 10.1056/NEJMoa2035389 PMC7787219 33378609

[3] Polack FP Thomas SJ Kitchin N Absalon J Gurtman A Lockhart S. Safety and Efficacy of the BNT162b2 Mrna Covid-19 Vaccine. N Engl J Med (2020) 383(27):2603–15. 10.1056/NEJMoa2034577 PMC7745181 33301246

[4] Piccoli L Park YJ Tortorici MA Czudnochowski N Walls AC Beltramello M. Mapping Neutralizing and Immunodominant Sites on the SARS-CoV-2 Spike Receptor-Binding Domain by Structure-Guided High-Resolution Serology. Cell (2020) 183(4):1024–42. 10.1016/j.cell.2020.09.037 PMC7494283 32991844

[5] Yuan M Liu H Wu NC Wilson IA. Recognition of the SARS-CoV-2 Receptor Binding Domain by Neutralizing Antibodies. Biochem Biophys Res Commun (2021) 538:192–203. 10.1016/j.bbrc.2020.10.012 33069360 PMC7547570

[6] Krissinel E Henrick K. Inference of Macromolecular Assemblies From Crystalline State. J Mol Biol (2007) 372(3):774–97. 10.1016/j.jmb.2007.05.022 17681537

[7] Raybould MIJ Kovaltsuk A Marks C Deane CM. CoV-AbDab: The Coronavirus Antibody Database. Bioinformatics (2020). 10.1093/bioinformatics/btaa739 PMC7558925 32805021

[8] Wu Y Wang F Shen C Peng W Li D Zhao C. A Noncompeting Pair of Human Neutralizing Antibodies Block COVID-19 Virus Binding to its Receptor ACE2. Science (2020) 368(6496):1274–8. 10.1126/science.abc2241 PMC7223722 32404477

[9] Du S Cao Y Zhu Q Yu P Qi F Wang G. Structurally Resolved SARS-Cov-2 Antibody Shows High Efficacy in Severely Infected Hamsters and Provides a Potent Cocktail Pairing Strategy. Cell (2020) 183(4):1013–23. 10.1016/j.cell.2020.09.035 PMC7489885 32970990

[10] Robbiani DF Gaebler C Muecksch F Lorenzi JCC Wang Z Cho A. Convergent Antibody Responses to SARS-CoV-2 in Convalescent Individuals. Nature (2020) 584(7821):437–42. 10.1038/s41586-020-2456-9 PMC7442695 32555388

[11] Barnes CO West AP Jr Huey-Tubman KE Hoffmann MAG Sharaf NG Hoffman PR. Structures of Human Antibodies Bound to SARS-CoV-2 Spike Reveal Common Epitopes and Recurrent Features of Antibodies. Cell (2020) 182(4):828–42. 10.1101/2020.05.28.121533 PMC7311918 32645326

[12] Clark SA Clark LE Pan J Coscia A McKay LGA Shankar S. Molecular Basis for a Germline-Biased Neutralizing Antibody Response to SARS-CoV-2. (2020). 10.1101/2020.11.13.381533

[13] Shi R Shan C Duan X Chen Z Liu P Song J. A Human Neutralizing Antibody Targets the Receptor-Binding Site of SARS-Cov-2. Nature (2020) 584(7819):120–4. 10.1038/s41586-020-2381-y 32454512

[14] Lu R Zhao X Li J Niu P Yang B Wu H. Genomic Characterisation and Epidemiology of 2019 Novel Coronavirus: Implications for Virus Origins and Receptor Binding. Lancet (2020) 395(10224):565–74. 10.1016/S0140-6736(20)30251-8 PMC7159086 32007145

[15] Wu NC Yuan M Liu H Lee CD Zhu X Bangaru S. An Alternative Binding Mode of IGHV3-53 Antibodies to the SARS-CoV-2 Receptor Binding Domain. Cell Rep (2020) 33(3):108274. 10.1016/j.celrep.2020.108274 33027617 PMC7522650

[16] Hurlburt NK Seydoux E Wan YH Edara VV Stuart AB Feng J. Structural Basis for Potent Neutralization of SARS-CoV-2 and Role of Antibody Affinity Maturation. Nat Commun (2020) 11(1):5413. 10.1038/s41467-020-19231-9 33110068 PMC7591918

[17] Lv Z Deng YQ Ye Q Cao L Sun CY Fan C. Structural Basis for Neutralization of SARS-CoV-2 and SARS-CoV by a Potent Therapeutic Antibody. Science (2020) 369(6510):1505–9. 10.1126/science.abc5881 PMC7402622 32703908

[18] Huo J Le Bas A Ruza RR Duyvesteyn HME Mikolajek H Malinauskas T. Neutralizing Nanobodies Bind SARS-CoV-2 Spike RBD and Block Interaction With ACE2. Nat Struct Mol Biol (2020) 27(9):846–54. 10.1038/s41594-020-0469-6 32661423

[19] Ju B Zhang Q Ge J Wang R Sun J Ge X. Human Neutralizing Antibodies Elicited by SARS-CoV-2 Infection. Nature (2020) 584(7819):115–9. 10.1038/s41586-020-2380-z 32454513

[20] Hansen J Baum A Pascal KE Russo V Giordano S Wloga E. Studies in Humanized Mice and Convalescent Humans Yield a SARS-CoV-2 Antibody Cocktail. Science (2020) 369(6506):1010–4. 10.1126/science.abd0831 PMC7299284 32540901

[21] Tortorici MA Beltramello M Lempp FA Pinto D Dang HV Rosen LE. Ultrapotent Human Antibodies Protect Against SARS-CoV-2 Challenge *Via* Multiple Mechanisms. Science (2020) 370(6519):950–7. 10.1126/science.abe3354 PMC7857395 32972994

[22] Liu L Wang P Nair MS Yu J Rapp M Wang Q. Potent Neutralizing Antibodies Against Multiple Epitopes on SARS-CoV-2 Spike. Nature (2020) 584(7821):450–6. 10.1038/s41586-020-2571-7 32698192

[23] Barnes CO Jette CA Abernathy ME Dam K-MA Esswein SR Gristick HB. Structural Classification of Neutralizing Antibodies Against the SARS-CoV-2 Spike Receptor-Binding Domain Suggests Vaccine and Therapeutic Strategies. (2020). 10.1101/2020.08.30.273920

[24] Cao Y Su B Guo X Sun W Deng Y Bao L. Potent Neutralizing Antibodies Against SARS-CoV-2 Identified by High-Throughput Single-Cell Sequencing of Convalescent Patients’ B Cells. Cell (2020) 182(1):73–84. 10.1016/j.cell.2020.05.025 32425270 PMC7231725

[25] Kreye J Reincke SM Kornau HC Sánchez-Sendin E Corman VM Liu H. A Therapeutic non-Self-Reactive SARS-CoV-2 Antibody Protects From Lung Pathology in a COVID-19 Hamster Model. Cell (2020) 183(4):1058–69. 10.1016/j.cell.2020.09.049 PMC7510528 33058755

[26] Liu H Wu NC Yuan M Bangaru S Torres JL Caniels TG. Cross-Neutralization of a SARS-CoV-2 Antibody to a Functionally Conserved Site is Mediated by Avidity. Immunity (2020) 53(6):1272–80. 10.1016/j.immuni.2020.10.023 PMC7687367 33242394

[27] Yuan M Wu NC Zhu X Lee CD So RTY Lv H. A Highly Conserved Cryptic Epitope in the Receptor Binding Domains of SARS-CoV-2 and SARS-Cov. Science (2020) 368(6491):630–3. 10.1126/science.abb7269 PMC7164391 32245784

[28] Zhou D Duyvesteyn HME Chen CP Huang CG Chen TH Shih SR. Structural Basis for the Neutralization of SARS-CoV-2 by an Antibody From a Convalescent Patient. Nat Struct Mol Biol (2020) 27(10):950–8. 10.1038/s41594-020-0480-y 32737466

[29] Pinto D Park YJ Beltramello M Walls AC Tortorici MA Bianchi S. Cross-Neutralization of SARS-CoV-2 by a Human Monoclonal SARS-CoV Antibody. Nature (2020) 583(7815):290–5. 10.1038/s41586-020-2349-y 32422645

[30] Wang P Nair MS Liu L Iketani S Luo Y Guo Y. Antibody Resistance of SARS-CoV-2 Variants B.1.351 and B.1.1.7. Nature (2021). 10.1038/s41586-021-03398-2 33684923

[31] Lan J Ge J Yu J Shan S Zhou H Fan S. Structure of the SARS-CoV-2 Spike Receptor-Binding Domain Bound to the ACE2 Receptor. Nature (2020) 581(7807):215–20. 10.1038/s41586-020-2180-5 32225176

[32] Huo J Zhao Y Ren J Zhou D Duyvesteyn HME Ginn HM. Neutralization of SARS-CoV-2 by Destruction of the Prefusion Spike. Cell Host Microbe (2020) 28(3):445–54. 10.1016/j.chom.2020.06.010 PMC7303615 32585135

[33] Weinreich DM Sivapalasingam S Norton T Ali S Gao H Bhore R. Regn-COV2, a Neutralizing Antibody Cocktail, in Outpatients With Covid-19. N Engl J Med (2020) 384(3):238–51. 10.1056/NEJMoa2035002 PMC7781102 33332778

[34] du Plessis L McCrone JT Zarebski AE Hill V Ruis C Gutierrez B. Establishment and Lineage Dynamics of the SARS-CoV-2 Epidemic in the UK. Science (2021) 371(6530):708–12. 10.1126/science.abf2946 PMC7877493 33419936

[35] Tegally H Wilkinson E Lessells RJ Giandhari J Pillay S Msomi N. Sixteen Novel Lineages of SARS-CoV-2 in South Africa. Nat Med (2021) 27(3):440–6. 10.1038/s41591-021-01255-3 33531709

[36] Starr TN Greaney AJ Addetia A Hannon WW Choudhary MC Dingens AS. Prospective Mapping of Viral Mutations That Escape Antibodies Used to Treat COVID-19. Science (2021) 371(6531):850–4. 10.1126/science.abf9302 PMC7963219 33495308

[37] Greaney AJ Loes AN Crawford KHD Starr TN Malone KD Chu HY. Comprehensive Mapping of Mutations in the SARS-CoV-2 Receptor-Binding Domain That Affect Recognition by Polyclonal Human Plasma Antibodies. Cell Host Microbe (2021) 29(3):463–76.e6. 10.1016/j.chom.2021.02.003 33592168 PMC7869748

